# Circular RNA_0000326 promotes bladder cancer progression via microRNA-338-3p/ETS Proto-Oncogene 1/phosphoinositide-3 kinase/Akt pathway

**DOI:** 10.1080/21655979.2021.2008738

**Published:** 2021-12-10

**Authors:** Yong Chen, Dong Wang, Tao Shu, Kangwei Sun, Jianbo Zhao, Min Wang, Yi Huang, Ping Wang, Hang Zheng, Zhixuan Cai, Zengyue Yang

**Affiliations:** Department of Urology Surgery, Xi’an International Medical Center Hospital, Xi’an, China

**Keywords:** Circ_0000326, bladder cancer, miR-338-3p, ETS1, PI3K/AKT

## Abstract

Circular RNAs (circRNAs) play a pivotal regulatory role in bladder cancer (BC) occurrence and progression. The expression level, role and mechanism of circ_0000326 in BC remain unknown. In the present study, quantitative reverse transcription-polymerase chain reaction (qRT-PCR) was conducted to evaluate the expressions of circ_0000326, microRNA-338-3p (miR-338-3p) and ETS Proto-Oncogene 1(ETS1) mRNA in BC tissues and cell lines. Cell counting kit-8 (CCK-8) assay, wound healing assay and flow cytometry were used to detect the impacts of circ_0000326 on BC cell growth, migration and apoptosis. Western blot was used to detect the expressions of ETS1, phospho-phosphoinositide-3 kinase (p-PI3K), phospho-AKT, PI3K and AKT protein. Gene ontology (GO) analysis and Kyoto Encyclopedia of Genes and Genomes (KEGG) pathway analysis were performed to analyze the biological function of ETS1 in BC. Here, we found that circ_0000326 expression was significantly elevated in BC cell lines and tissues, and circ_0000326 could promote BC cell growth and migration, and inhibit apoptosis. Dual-luciferase reporter gene assay confirmed that circ_0000326 and ETS1 could bind directly to miR-338-3p. Furthermore, circ_0000326 sponged miR-338-3p and up-regulated ETS1 expression. ETS1 was associated with the activation of PI3K/AKT pathway. Moreover, circ_0000326 could activate PI3K/AKT pathway by miR-338-3p/ETS1 axis. Collectively, circ_0000326/miR-338-3p/ETS1/PI3K/AKT pathway is involved in regulating BC progression.

## Introduction

Bladder cancer (BC) is known as a common urological malignancy. Reportedly, there were 80,500 new cases and 32,900 death cases in China in 2015, and the morbidity and mortality have been increasing year by year [1]. Specifically, BC’s recurrence rate within 5 years is as high as 70% [2]. Despite dramatic advances in surgery and chemotherapy for BC, it is still necessary to unearth novel biomarkers and treatment targets for BC.

Circular RNAs (circRNAs) are featured with a covalent closed-loop structure without 3ʹ and 5ʹ ends, with higher stability and abundance compared with linear RNAs [3]. CircRNAs are derived from the ‘direct reverse splicing’ or ‘exon skipping’ of pre-mRNA transcripts, and they were previously thought to be junk products of splicing errors. Nonetheless, in recent years, a lot of studies have reported that circRNAs contribute to regulating gene expression, and one of their roles is to act as competing endogenous RNAs (ceRNAs) of miRNAs [4]. For example, in BC, circMBOAT2 promotes the proliferation and migration of BC cells by regulating miR-433-3p/CREB1 axis [5]. CircZFR promotes the proliferation, migration and invasion of BC cells by upregulating WNT5A expression via sponging miR-545 and miR-1270 [6]. Circ_0001495 promotes the proliferation, migration and invasion of BC cells *in vitro* through miR-527/Robo1 axis [7]. By analyzing GSE92675 from the GEO database, in this study, we found that circ_0000326 expression was up-regulated in BC tissues compared with adjacent tissues. Previous studies also show that circ_0000326 acts as an oncogene in some malignancies, such as lung cancer and cervical cancer [8,9]. However, the role of circ_0000326 in BC remains unknown.

MicroRNAs (miRNAs or miRs) can modulate gene expression by inhibiting mRNA translation. MiRNAs, reportedly, are involved in various biological processes including cell proliferation, apoptosis and migration [10]. Various miRNAs are aberrantly expressed in many malignancies including BC. For example, miR-338-3p can bind to ETS Proto-Oncogene 1 (ETS1) and play an essential role in the tumorigenesis of BC [11]. However, the mechanism of the dysregulation of miR-338-3p/ETS1 axis and the downstream of EST1 in BC remain unclear.

We hypothesized that the dysregulation of circ_0000326 expression might contribute to the development and progression of BC through the regulation of miR-338-3p/ETS1 axis. In the present study, we investigated the expression and the role of circ_0000326 in BC cells. Moreover, the regulatory relationship between circ_0000326 and miR-338-3p/ETS1/PI3K/AKT axis was studied.

## Methods

### *Clinical sample collection* [12]

From 2020 to 2021, tumorous and para-cancerous tissues surgically removed from 70 BC patients were collected, and then the tissues were immediately snap-frozen using liquid nitrogen and stored at −80°C. This study was endorsed by the Ethics Committee of Xi’an International Medical Center Hospital (No. 2,020,079) (Supplementary file), and all subjects offered a signed informed consent prior to surgery.

### *Cell culture* [12]

Human bladder epithelial cell line (SV-HUC-1) and BC cell lines (BIU-87, 5637, RT-112 and T24) were obtained at the Cell Bank of the Chinese Academy of Sciences (Shanghai, China). All cells were cultured in a Roswell Park Memorial Institute (RPMI)-1640 medium (Gibco, Carlsbad, California, USA) supplemented with 10% fetal bovine serum (FBS; Gibco, Carlsbad, California, USA), streptomycin (100 μg/mL; Gibco, Carlsbad, California, USA) and penicillin (100 U/mL; Gibco, Carlsbad, California, USA) at 37°C in 5% CO_2_.

### *Cell transfection* [12]

Circ_0000326 small interfering RNA (siRNA), scrambled siRNA, miR-338-3p inhibitor, miR-338-3p mimics and their negative control, ETS1 overexpression plasmid and empty plasmid were all purchased from Genomeditech (Shanghai, China). Lipofectamine^TM^ 2000 (Invitrogen, Carlsbad, CA, USA) was adopted to transfect the above oligonucleotides/vectors into BC cell lines.

### *Quantitative real-time polymerase chain reaction (qRT-PCR)* [12]

Total RNA from tissues and cell lines was extracted with TRIzol reagent (Invitrogen, Carlsbad, CA, USA). Subsequently, a Prime Script RT Master Mix kit (Takara, Japan) was used to synthesize 1 μg of RNA into complementary DNA. Next, qRT-PCR was conducted with a SYBR Green Premix Ex Taq^TM^ kit (Takara, Japan). U6 acted as an internal reference for miRNA, and GAPDH acted as an internal reference for circ_0000326 and ETS1. Below are the primer sequences (F: forward; R: reverse): circ_0000326, 5ʹ-TTGAATAGATTTCAGCTTTATGC-3ʹ (F) and 5ʹ-CCCATAACTGATCTGACTTTGT-3ʹ (R); miR-338-3p, 5ʹ-GGCGGAGTTTATGGGTTTTC-3ʹ (F) and 5ʹ-AAACCTAACCGATCCTCG-3ʹ (R); ETS1: 5ʹ-AGGGACAGAGCGGAACTCAAC-3ʹ (F) and 5ʹ-AATTGGTCCGCTTCCTGTGTAG-3ʹ (R); MALAT1, 5ʹ-GAATTGCGTCATTTAAAGCCTAGTT-3ʹ (F), 5ʹ-GTTTCATCCTACCACTCCCAATTAAT-3ʹ (R); U6, 5ʹ-GACTATCATATGCTTACCGT-3ʹ (F) and 5ʹ-GGGCAGGAAGAGGGCCTAT-3ʹ (R); GAPDH, 5ʹ-CTCTGCTCCTCCTGTTCGAC-3ʹ (F) and 5ʹ-CGACCAAATCCGTTGACTCC-3ʹ (R).

### *RNase R (3ʹ-5ʹ exoribonuclease) treatment* [13]

The extracted RNA from the cells was separated into two groups. One group was treated with RNase R (Geneseed, Guangzhou, China) for 30 min, and the other group served as the control. Then, the expression levels of circ_0000326 and MALAT1 (a linear non-coding RNA) in the two groups were detected by qRT-PCR.

### *Cell proliferation assay* [13]

Cell proliferation was measured by cell counting kit-8 (CCK-8; Genomeditech, Shanghai, China) assay. The transfected BC cells were inoculated at 5 × 10^3^ cells/well into 96-well plates. At 0, 24, 48 and 72 h, each well was added with CCK-8 reagent (10 µL), with which the cells were incubated for 2 h. Next, the absorbance at 450 nm wavelength was measured by a microplate reader (Thermo-Fisher Scientific, Waltham, MA, USA).

### *Wound healing assay* [13]

BC cells in the logarithmic growth phase were inoculated in 6-well plates (1 × 10^6^ cells/well). After the cells spread over the bottom of the wells, the medium was replaced with cell culture medium with 1% FBS. Then, the cells were cultured for 12 h, and a pipette tip (200 μL) was employed to draw a line through the center of each well. At 0 and 24 h, the photos of the scratches were captured. Wound width closure (%) = (0 h scratch width – 24 h scratch width/0 h scratch width) × 100%.

### *Dual-luciferase reporter assay* [13]

Wild-type (WT) and mutant-type (MUT) circ_0000326 sequence or ETS1 3ʹ-UTR sequence containing the binding site of miR-338-3p was inserted into luciferase reporter plasmids (Sangon Biotech,Shanghai,China).Subsequently, Lipofectamine^TM^ 2000 was used to co-transfect the above luciferase reporters and miR-338-3p mimics or miR-NC into BC cells. After 24 h, the Dual-Luciferase Reporter Assay System (Beyotime, Shanghai, China) was utilized to detect the luciferase activity.

### *RNA immunoprecipitation (RIP) assay* [14]

The RIP assay Kit (Thermo Fisher Scientific, MA, USA) was used to perform the RIP assay. Briefly, the cells were lysed in RIP buffer, and incubated with the magnetic beads conjugated with Argonaute 2 antibody (anti-Ago2) or immunoglobulin G antibody (anti-IgG; control) at 4 C overnight. Subsequently, RNA was eluted and purified, and immunoprecipitated RNA was analyzed by qRT-PCR to detect the enrichment of miR-338-3p and ETS1 mRNA.

### *Western blot* [14]

The radio-immunoprecipitation assay (RIPA) lysis buffer (Beyotime, Shanghai, China) was employed for protein extraction from tissues and cells. The supernatant was collected after centrifugation. The bicinchoninic acid (BCA) protein assay kit (Thermo-Fisher Scientific, Waltham, MA, USA) was employed to detect protein concentration. Subsequently, proteins (40 μg/well) were separated by sodium dodecyl sulfate-polyacrylamide gel electrophoresis and transferred to polyvinylidene difluoride membranes (Millipore, Billerica, MA, USA). After that, the membranes were blocked in 5% skim milk and incubated overnight with primary antibodies [anti-GAPDH: 1:1000, ab9485, Abcam Inc., Cambridge, UK; anti-ETS1: 1:500, ab220361, Abcam Inc., Cambridge, UK, anti-phospho-PI3K (p-PI3K): 1:1000, #17,366, Cell Signaling Technology, Massachusetts, USA; anti-p-AKT: 1:1000, #4060, Cell Signaling Technology, Massachusetts, USA; anti-PI3K: 1:1000, #4292, Cell Signaling Technology, Massachusetts, USA; anti-AKT: 1:1000, #9272, Cell Signaling Technology, Massachusetts, USA] at 4°C. Next, the membranes were incubated with horseradish peroxidase-conjugated secondary antibody (1:2000, ab150077, Abcam Inc., Cambridge, UK) for 2 h at room temperature. Ultimately, protein bands were visualized by the ECL luminescence reagent (Invitrogen; Thermo Fisher Scientific, Inc., Waltham, MA, USA), with GAPDH as the internal reference.

### *Flow cytometry analysis* [15]

The apoptosis of BC cells was detected by annexin V and propidium iodide (PI) staining assay. To be specific, the cells were treated with 0.25% trypsin, and then resuspended in a binding buffer. Then, for each sample, the cell suspension was incubated with 5 µL of fluorochrome-conjugated annexin V staining solution (Sigma-Aldrich, Saint Louis, USA) for 30 min in the dark. Subsequently, 5 µL of PI staining solution (Sigma-Aldrich, Saint Louis, USA) was added to stain the cells for 30 min in the darkness. The percentage of apoptotic cells was detected with a flow cytometer (FACS Calibur, Becton Dickinson, and Sunnyvale, CA, USA).

### *Bioinformatics analysis* [16,17]

The circRNA expression profile dataset GSE92675 was obtained from the GEO database (http://www.ncbi.nlm.nih.gov/geo/). The differentially expressed circRNAs were screened by GEO2R, and *P* value <0.05 and|log2 fold change (FC) | >2 were set as the thresholds. Gene expression profiling interactive analysis (GEPIA) (http://gepia.cancer-pku.cn/) is a web tool for analyzing RNA sequencing data from the TCGA database in the standard processing pipeline. Genes that were correlated with ETS1 in BC were obtained using GEPIA. The thresholds are defined as the Pearson’s correlation coefficient R ≥ 0.5. Then, gene Ontology (GO) classification and Kyoto Encyclopedia of Genes and Genomes (KEGG) pathway enrichment analysis were performed using the DAVID database (https://david.ncifcrf.gov/).

### *Statistical analysis* [17]

Each experiment was performed in triplicate and repeated 3 times, with ‘mean ± standard deviation’ representing the results. SPSS 17.0 (SPSS Inc., Chicago, IL, USA) was a statistical analysis tool. Comparison between two groups and among multiple groups was performed via Student’s *t*-test and one-way analysis of variance, respectively, and correlation analysis was conducted by Pearson’s correlation analysis. Chi-square test was used to analyze the association between the expression level of circ_0000326 and pathological characteristics. If *P* < 0.05, the difference was of statistical significance.

## Results

This study was performed to explore the function and mechanism of circ_0000326 in BC development, and it was demonstrated that that circ_0000326 expression was upregulated in BC tissues and cell lines. *In vitro* experiments showed that circ_0000326 could promote BC cell growth and migration, and inhibit cell apoptosis. Mechanistically, circ_0000326 activated the PI3K/AKT signal pathway by sponging miR-338-3p and upregulating the expression of ETS1 (Graphic Abstract).

### Circ_0000326 expression in BC was significantly up-regulated

**Figure 1.**
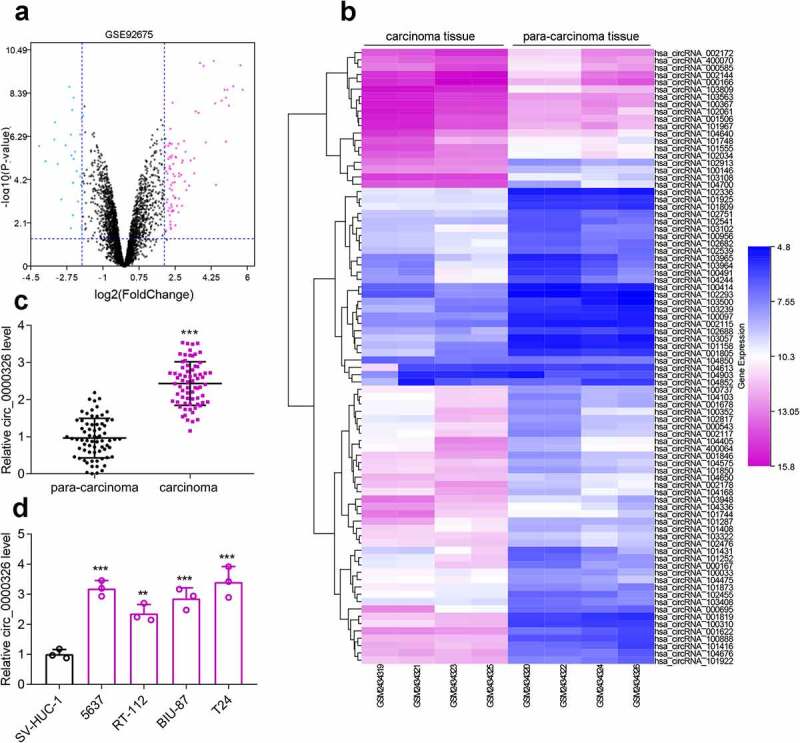
Circ_0000326 expression was significantly up-regulated in BC. (a). The volcano plot of differentially expressed genes (DECs) in GSE92675. The blue and purple dots represented the downregulated DECs and upregulated DECs with statistical significance (log2 |fold change|>2 and *P* < 0.05), respectively. (b). The heatmap of DECs whose expressions were upregulated in GSE92675.C-D. qRT-PCR showed that circ_0000326 expression was upregulated in BC tissues and cell lines.***P < 0.001

Microarray analysis was conducted to identify differentially expressed circRNAs in BC tissues and para-cancerous tissues. According to cutoff criteria (log2 |fold change|>2 and *P* < 0.05), it was revealed that circ_000543 (circ_0000326) expression in BC tissues was markedly higher than that in para-cancerous tissues (Figure 1(a-b)). qRT-PCR showed that circ_0000326 expression in 70 cases of BC tissues was remarkably higher as opposed to that in normal tissues (Figure 1(c)). Additionally, circ_0000326 expression in BC cell lines (5637, RT-112, BIU-87, and T24), compared to that in the SV-HUC-1 cell line, was significantly higher (Figure 1(d)). Subsequently, with the median expression level of circ_0000326 as the cutoff value, BC patients were divided into high (n = 35) and low (n = 35) expression groups. Then the relationship between circ_0000326 expression and BC clinicopathological features was analyzed, and it was revealed that highly expressed circ_0000326 was associated with relatively larger tumor diameter and advanced clinical stage (Table 1). Hence, our findings indicated that circ_0000326 expression was significantly enhanced in BC tissues, and circ_0000326 was likely to participate in BC progression as a tumor-promoting circRNA.

**Table 1.** Correlation between circ_0000326 expression and clinicopathological parameters

Parameter	n	circ_0000326		*P*
		High (n = 35)	Low (n = 35)	
Age (years)				
≥60	30	13	17	0.336
< 60	40	22	18	
Gender				
Male	36	21	15	0.152
Female	34	14	20	
Tumor size (cm)				
≥3	40	25	15	0.015
<3	30	10	20	
Clinical stage				
III-IV	34	22	12	0.016
I-II	36	13	23	
Lymphatic metastasis				
Negative	34	18	16	0.63
Positive	36	17	19

### Circ_0000326 promoted BC cell growth and migration, and inhibited apoptosis

**Figure 2.**
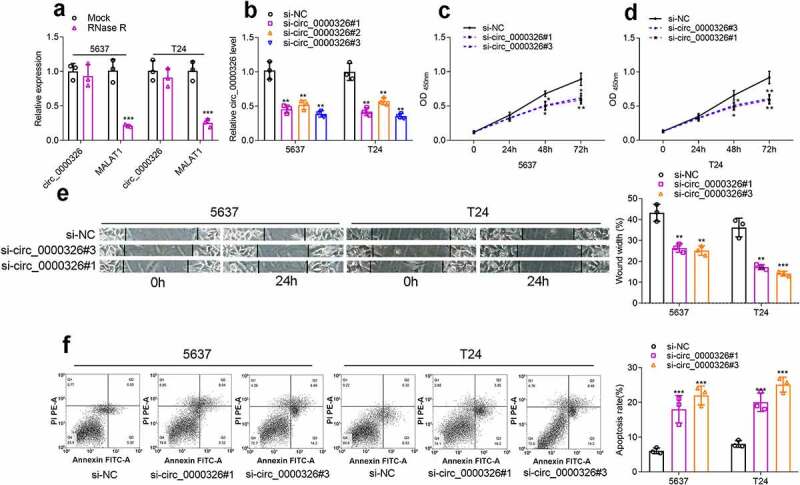
Circ_0000326 promoted BC cell proliferation and migration, and inhibited the apoptosis. (a). qRT-PCR showed that linear MALAT1 was degraded by RNase R, while circ_0000326 could not be degraded by RNase R. (b). qRT-PCR showed that circ_0000326 expression was inhibited in BC cells transfected with circ_0000326 siRNAs. (c-d). CCK-8 assay showed that cell proliferation in the si-circ_0000326#1 and si-circ_0000326#3 groups was dramatically suppressed as against the si-NC group. (e). Would healing assay indicated that in comparison to the control group, cell migration capacity in the si-circ_0000326#1 and si-circ_0000326#3 groups was significantly reduced. (f). Flow cytometry showed that the apoptosis rate was significantly increased after circ_0000326 knockdown.*P < 0.05, **P < 0.01 and ***P < 0.001

To detect the circular structure of circ_0000326, RNA was extracted from BC cells, and then treated with RNase R, and the results of qRT-PCR confirmed that linear MALAT1 was degraded, while circ_0000326 could not be degraded by RNase R (Figure 2(a)). Next, to further identify the regulatory effects of circ_0000326 on the biological behaviors of BC cells, three kinds of circ_0000326 siRNAs were transfected into two BC cell lines (T24 and 5637) to construct the circ_0000326 knockdown model. It was revealed that the si-circ_0000326#1 and si-circ_0000326#3 showed better knockdown efficiency, so these two siRNAs were selected for subsequent experiments (Figure 2(b)). CCK-8 assay was conducted to detect cell proliferation, and it was confirmed that the cell proliferation ability in the si-circ_0000326#1 and si-circ_0000326#3 groups was observably lower than that in si-NC group (Figure 2(c-d)). Wound healing assay was performed to examine cell migration capability, and it was revealed that cell migration capacity in the si-circ_0000326#1 and si-circ_0000326#3 groups was significantly reduced in comparison to that in the control group (Figure 2(e)). Furthermore, flow cytometry showed that the apoptosis rate of the transfected BC cells was significantly increased after circ_0000326 knockdown (figure 2(f)). Therefore, the above-mentioned data confirmed that circ_0000326 participated in regulating the malignant phenotype of BC cells.

### Circ_0000326 could sponge miR-338-3p

**Figure 3.**
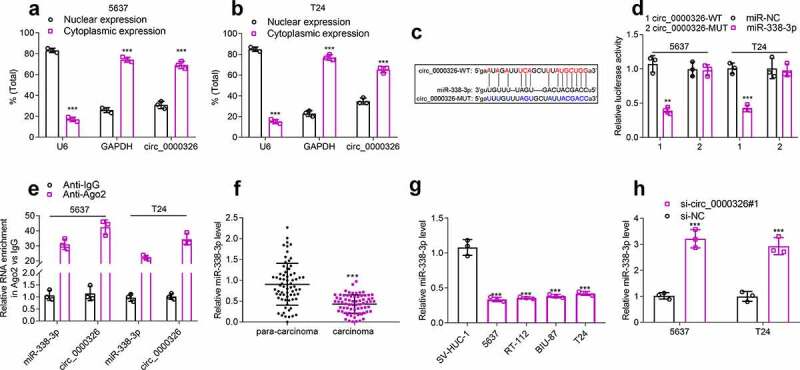
Circ_0000326 could combine with miR-338-3p in BC. (a-b). qRT-PCR indicated that circ_0000326 was mainly distributed in the cytoplasm of 5637 and T24 cell lines. (c). StarBase database was utilized to predict the binding site between circ_0000326 and miR-338-3p. (d). Dual-luciferase reporter assays showed that the luciferase activity of wild-type circ_0000326 reporter was significantly suppressed by miR-338-3p mimics, while the luciferase activity of the mutated reporter was not affected by miR-338-3p mimics. (e). RIP assay showed that circ_0000326 and miR‐338-3p were mainly enriched by anti-Ago2 antibody. (f-g). qRT-PCR showed that miR-338-3p expression was markedly down-regulatedin BC cell lines and tissues. (h). qRT-PCR showed that miR-338-3p expression was significantly increased in BC cells transfected with circ_0000326 siRNA.**P < 0.01 and ***P < 0.001

The cytoplasm and nuclei of BC cells were then separated to determine the distribution of circ_0000326. qRT-PCR indicated that circ_0000326 was mainly distributed in the cytoplasm of 5637 and T24 cell lines (Figure 3(a-b)). So, circ_0000326 might participate in BC progression at the post-transcriptional level, and circ_0000326 was likely to exert its biological functions as a ceRNA in BC. StarBase database showed that miR-338-3p contained the binding site complementary to circ_0000326 (Figure 3(c)). To further verify the binding relationship between circ_0000326 and miR-338-3p, we constructed a circ_0000326 wild-type luciferase reporter vector containing the binding sequence of miR-338-3p and a mutant luciferase reporter vector in which the binding sequence was mutated (Figure 3(c)). Dual-luciferase reporter assay showed that luciferase activity of the wild-type reporter was significantly suppressed by miR-338-3p mimics, while that of the MUT luciferase reporter was not affected by miR-338-3p mimics (Figure 3(d)). Moreover, RIP assay showed that circ_0000326 and miR‐338-3p were mainly enriched by anti-Ago2 antibody (Figure 3(e)). Subsequently, qRT-PCR was performed for detecting miR-338-3p expression in BC cell lines and tissues, and it was revealed that miR-338-3p expression was markedly down-regulated in BC cell lines and tissues (figure 3(f-g)). Notably, after circ_0000326 knockdown, miR-338-3p expression in 5637 and T24 cell lines was significantly increased (Figure 3(h)). These data suggested that in BC cells, circ_0000326 could sponge miR-338-3p to repress its expression.

### Circ_0000326 could target miR-338-3p to modulate ETS1 expression

**Figure 4.**
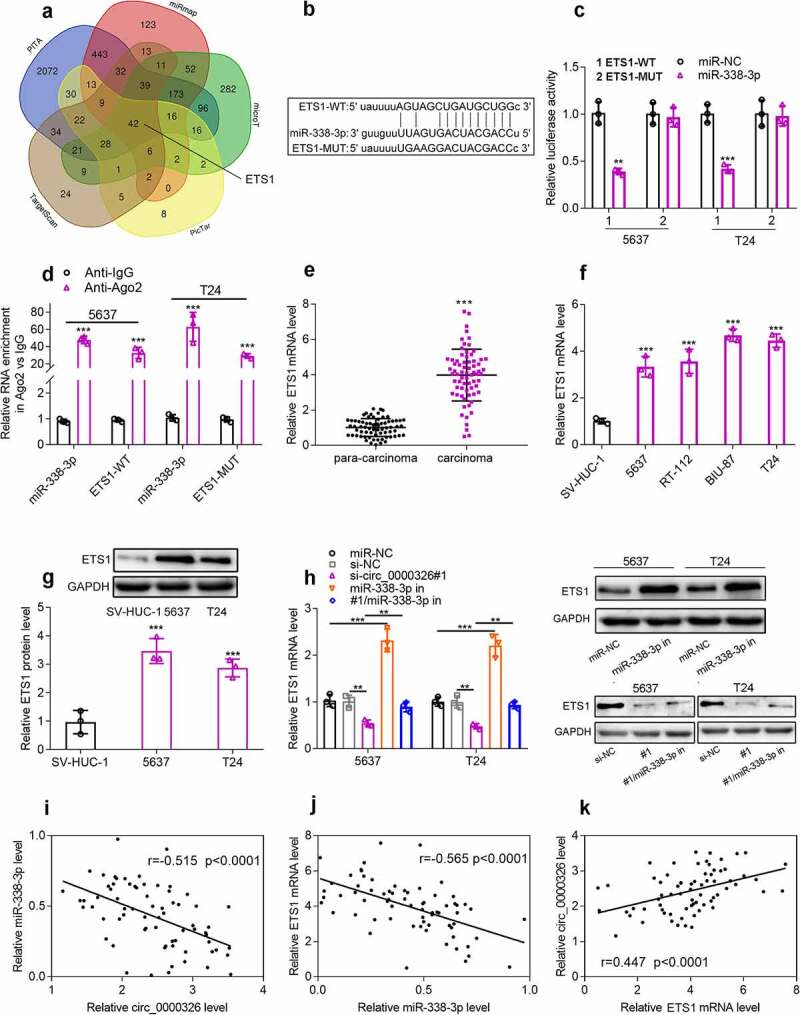
Circ_0000326 could target miR-338-3p to regulate ETS1 expression

Next, TargetScan, PicTar, PITA miRmap, microT and miRDB databases were applied to pinpoint the downstream targets of miR-338-3p, and ETS1 was among the downstream targets predicted by all the four databases (Figure 4(a-b)). Dual-luciferase reporter gene assay showed that miR-338-3p mimics could inhibit the luciferase activity of the wild-type ETS1 luciferase reporter vector but exert no impact on that of the mutant-type reporter (Figure 4(c)). Next, RIP assay was performed, and it showed that miR-338-3p and ETS1 mRNA were enriched in Anti-Ago2 group compared with Anti-IgG group, suggesting that miR-338-3p could interact with ETS1 mRNA in BC cells (Figure 4(d)). To elucidate whether circ_0000326 regulated ETS1 expression by repressing miR-338-3p, qRT-PCR and Western blot were conducted, and it was discovered that ETS1 expression was notably up-regulated in BC tissues and cells (Figure 4(e-g)); miR-338-3p inhibitor could significantly elevate ETS1 expression in BC cell lines, and miR-338-3p inhibitor counteracted the inhibitory effect of circ_0000326 knockdown on ETS1 expression (Figure 4(h)). Pearson correlation analysis confirmed that in BC tissues, circ_0000326 expression was negatively correlated with miR-338-3p expression and positively correlated with ETS1 mRNA expression, while miR-338-3p was negatively correlated with ETS1 expression (Figure 4(h-j)). Therefore, the above experiments implied that in BC cell lines, circ_0000326 could target miR-338-3p to regulate ETS1 expression.

(a). Venn diagram of mRNAs containing the potential binding site to miR-338-3p by searching TargetScan, PicTar, PITA miRmap, microT and miRDB databases. (b). StarBase database was employed to predict the binding site between miR-338-3p and ETS1. (c). Luciferase reporter assay confirmed that miR-338-3p could negatively regulate the luciferase activity of circ_0000326-WT, rather than circ_0000326-Mut. (d). RIP assay showed that miR-338-3p and ETS1 were all enriched in Anti-Ago2 group compared with Anti-IgG group. (e-g). qRT-PCR and Western blot showed that ETS1 expression was notably up-regulatedin BC tissues and cells. (h). qRT-PCR and Western blot showed that miR-338-3p inhibitor counteracted the inhibitory effect of circ_0000326 knockdown on ETS1 expression. (i-k). Pearson correlation analysis was used to analyze the relationships between circ_0000326 and miR-338-3p, miR-338-3p and ETS1 mRNA, as well as miR-338-3p and ETS1 mRNA in BC.**P < 0.01 and ***P < 0.001.

### Circ_0000326 could regulate miR-338-3p/ETS1 to participate in BC progression

**Figure 5.**
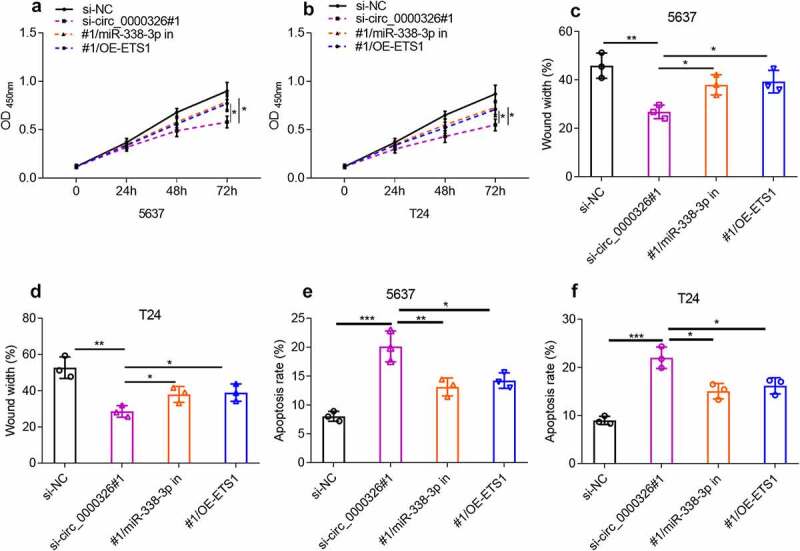
Circ_0000326 could regulate miR-338-3p/ETS1 to participate in BC progression. (a-b). CCK-8 assay was used to verify the cell proliferation in BC cells transfected with si-circ_0000326, circ_0000326 siRNA and miR-338-3p inhibitor, or circ_0000326 siRNA and ETS1 overexpression plasmid. (c-d). Wound healing assay was used to detect the migration of BC cells transfected with si-circ_0000326, circ_0000326 siRNA and miR-338-3p inhibitor, or circ_0000326 siRNA and ETS1 overexpression plasmid. (e-f). Flow cytometry was used to detect the apoptosis of BC cells transfected with si-circ_0000326, circ_0000326 siRNA and miR-338-3p inhibitor, or circ_0000326 siRNA and ETS1 overexpression plasmid. *P < 0.05, **P < 0.01 and ***P < 0.001

To verify that circ_0000326 participated in the BC development via modulating the miR-338-3p/ETS1 axis, circ_0000326 siRNA and miR-338-3p inhibitor, or circ_0000326 siRNA and ETS1 overexpression plasmid was co-transfected into 5637 and T24 cell lines, respectively. CCK-8 assay and wound healing assay confirmed that compared with the si-circ_0000326 group, the co-transfection of circ_0000326 siRNA and miR-338-3p inhibitor, or of circ_0000326 siRNA and ETS1 overexpression plasmid, could significantly enhance cell proliferation and migration (Figure 5(a-d)). Flow cytometry indicated that miR-338-3p inhibitor or ETS1 overexpression plasmid partly reversed the promoting effect of circ_0000326 knockdown on cell apoptosis (Figure 5(e-f)). These data implied that circ_0000326 exerted its biological function via miR-338-3p and ETS1.

### Circ_0000326/miR-338-3p/ETS1 activated PI3K/AKT pathway in BC

**Figure 6.**
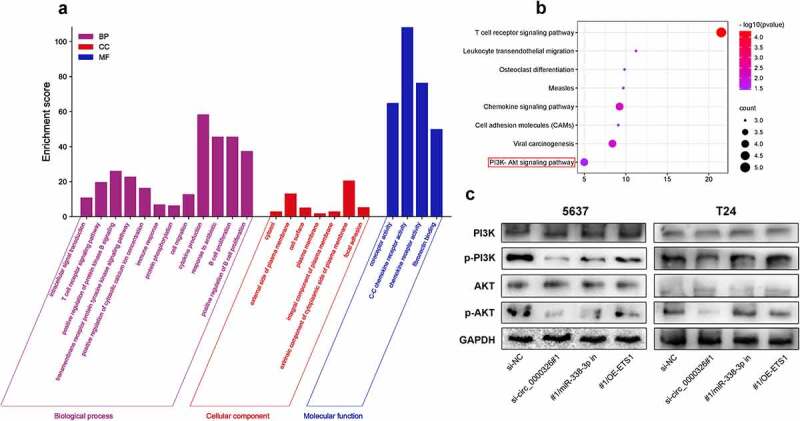
Circ_0000326/miR-338-3p/ETS1 activated PI3K/AKT signal pathway in BC

To elucidate the potential mechanism underlying ETS1 in BC progression, a total of 20 similar genes of ETS1 were utilized to perform the GO analysis (including biological processes, cellular component and molecular function terms) and KEGG pathway enrichment analysis. The most enriched biological process terms were ‘intracellular signal transduction’, ‘cell migration’ and ‘cytokine production’; the most enriched cellular component terms were ‘cytosol’ and ‘cell surface’; the most enriched molecular function terms were ‘coreceptor activity’ and ‘chemokine receptor activity’ (Figure 6(a)). In addition, KEGG analysis showed that ‘chemokine signaling pathway’ and ‘PI3K-Akt signal pathway’ were significantly enriched pathways (Figure 6(b)). We hypothesized that circ_0000326/miR-338-3p/ETS1 probably regulated PI3K/AKT signaling pathway in the progression of BC cells. Western blot confirmed that circ_0000326 knockdown could significantly reduce p-PI3K and p-Akt protein expression, and compared with the si-circ_0000326 group, the co-transfection of circ_0000326 siRNA and miR-338-3p inhibitor, or of circ_0000326 siRNA and ETS1 overexpression plasmid, could significantly restore the expressions of p-PI3K and p-Akt protein; however, the expressions of PI3K and Akt remained unchanged (Figure 6(c)).

(a-b): GO and KEGG pathway enrichment analyses were used to predict the biological function of ETS1 in BC. (c): Western blot was used to detect p-PI3K, p-AKT, PI3K and AKT protein expressions in BC cells transfected with si-circ_0000326, circ_0000326 siRNA and miR-338-3p inhibitor, or circ_0000326 siRNA and ETS1 overexpression plasmid.

## Discussion

Previous studies report that circRNAs are closely associated with BC occurrence and development [5–7]. Circ_0000326 is transcribed from chromosome 11: 65,272,490–65,272,586. It is reported that circ_0000326 is significantly upregulated in lung adenocarcinoma tissue, and it can target miR-338-3p to promote lung adenocarcinoma cell proliferation and migration, and inhibit cell apoptosis [9]. Circ_0000326 also exerts oncogenic effects in cervical cancer by upregulating cyclin-dependent kinase 4 via sponging miR-338-3p [8]. In this study, it was revealed that circ_0000326 expression was markedly up-regulated in BC tissues. Moreover, circ_0000326 expression was significantly higher in BC cell lines compared to normal human bladder epithelial cell line. Additionally, the high expression of circ_0000326 was associated with the unfavorably pathological characteristics of the patients. Subsequently, loss-of-function assays were performed to explore the biological functions of circ_0000326 in BC cell lines, and it was found that knocking down circ_0000326 could repress BC cell growth and migration, and inhibit apoptosis. The functions of circ_0000326 in BC are similar to the previous reports, showing that circ_0000326 exerted oncogenic effect [8,9].

It is well known that circRNAs can function as ceRNAs to regulate genes’ expression by targeting miRNAs [18]. Our findings suggested that circ_0000326 was distributed mainly in the cytoplasm of BC cells, so it was supposed that circ_0000326 might function as a ceRNA. Bioinformatics and luciferase reporter assays confirmed that miR-338-3p could directly bind to circ_0000326. As a tumor suppressor, miR-338-3p can inhibit the progression of a variety of tumors. For example, miR-338-3p expression is significantly reduced in multiple myeloma, and miR-338-3p can target BRD4 to suppress cell growth and migration, and facilitate apoptosis [19]. In breast cancer, miR-338-3p can negatively regulate ZEB2 to repress cell growth, migration, invasion and epithelial–mesenchymal transition (EMT) [20]. In colorectal cancer, miR-338-3p can down-regulate MACC1 expression to suppress cancer cell growth, migration and invasion [21]. Importantly, it is reported that in BC, miR-338-3p expression is significantly down-regulated, and miR-338-3p inhibits BC cell growth, metastasis and EMT [11]. Our findings suggest that miR-338-3p is a tumor suppressor in BC, which is consistent with the previous studies. Additionally, we found that miR-338-3p expression was negatively correlated with circ_0000326 expression, and knockdown of circ_0000326 in BC cell lines could markedly promote miR-338-3p expression. Furthermore, the co-transfection of miR-338-3p inhibitor could counteract the inhibiting effects of circ_0000326 knockdown on BC cell proliferation and migration, and the promoting effect on apoptosis. Therefore, it was concluded that circ_0000326 played a role in BC cells via modulating miR-338-3p expression.

Recognized as a transcription factor belonging to the ETS family, ETS1 can regulate immunity and angiogenesis [22]. ETS1 dysregulation is also associated with the tumorigenesis and progression of several malignancies. Previous research confirms that ETS1 overexpression can up-regulate the expression of matrix metalloproteinases to enhance the cancer cell migration and invasion in pancreatic, prostate, ovarian and colon cancers [22]. In renal carcinoma and glioma, ETS1 promotes cell proliferation by up-regulating TGFα expression; in hepatocellular carcinoma, ETS1 promotes cell proliferation by activating the expressions of cyclin E and cyclin-dependent kinase 2 (CDK2) [22]. In colorectal cancer and breast cancer, ETS1 can bind to the promoters of some apoptotic genes (such as CDKN1A (encoding p21 protein), CDKN1B (encoding p27 protein), TP53, caspase I, etc.) to regulate apoptosis [23,24]. Besides, miR-338-3p can regulate the expression of EST1 and partake in BC development [11]. Our study also found that ETS1 was the target gene of miR-338-3p, and its expression was markedly increased in BC cell lines and tissues; ETS1 expression was negatively correlated with miR-338-3p expression and positively linked to circ_0000326 expression. Moreover, it was revealed that circ_0000326 up-regulated ETS1 expression by inhibiting miR-338-3p expression. Additionally, ETS1 overexpression could counteract the effects of circ_0000326 knockdown on BC cell proliferation, migration and apoptosis. With these findings, it is concluded that, in BC, circ_0000326, miR-338-3p and ETS1 form a ceRNA network to regulate disease progression.

The PI3K/AKT pathway plays an important role in cancer development and is involved in the regulation of a variety of phenotypes, including proliferation, migration, differentiation and apoptosis [25]. The activation of PI3K/AKT pathway promotes the proliferation and inhibits the apoptosis and autophagy of BC cells [26]. The present study also demonstrated that circ_0000326 activated the PI3K/AKT pathway, and miR-338-3p inhibitor or ETS1 overexpression reversed the inhibitory effects of circ_0000326 knockdown on the PI3K/AKT pathway, indicating that circ_0000326 was involved in the regulation of BC progression through the miR-338-3p/ETS1/PI3K/AKT pathway.

## Conclusion

To sum up, circ_0000326 is highly expressed in BC tissues, and circ_0000326/miR-338-3p/ETS1/PI3K/AKT axis was involved in BC progression, and circ_0000326 may be a new target for BC diagnosis and treatment. However, the present study has certain limitations that need to be mentioned. First of all, the relationship between circ_0000326 expression and the prognosis of BC patients is still unclear. Moreover, the function of circ_0000326 in BC has not been confirmed via *in vivo* experiments.

## Supplementary Material

Supplemental Material

## Highlights

- Circ_0000326 expression was significantly upregulated in BC tissues and cell lines.
- Circ_0000326 promoted BC cell proliferation and migration, and inhibited cell apoptosis.
- Circ_0000326 activated PI3K/AKT signal pathway by sponging miR-338-3p and up-regulating ETS1 expression in BC.
