# Supplementary figures and images for: Circular RNA_0000326 promotes bladder cancer progression via microRNA-338-3p/ETS Proto-Oncogene 1/phosphoinositide-3 kinase/Akt pathway
Source: Bioengineered. 2021 Dec 10;12(2):11410–22. doi: 10.1080/21655979.2021.2008738 (PMC8810167; doi:10.1080/21655979.2021.2008738)

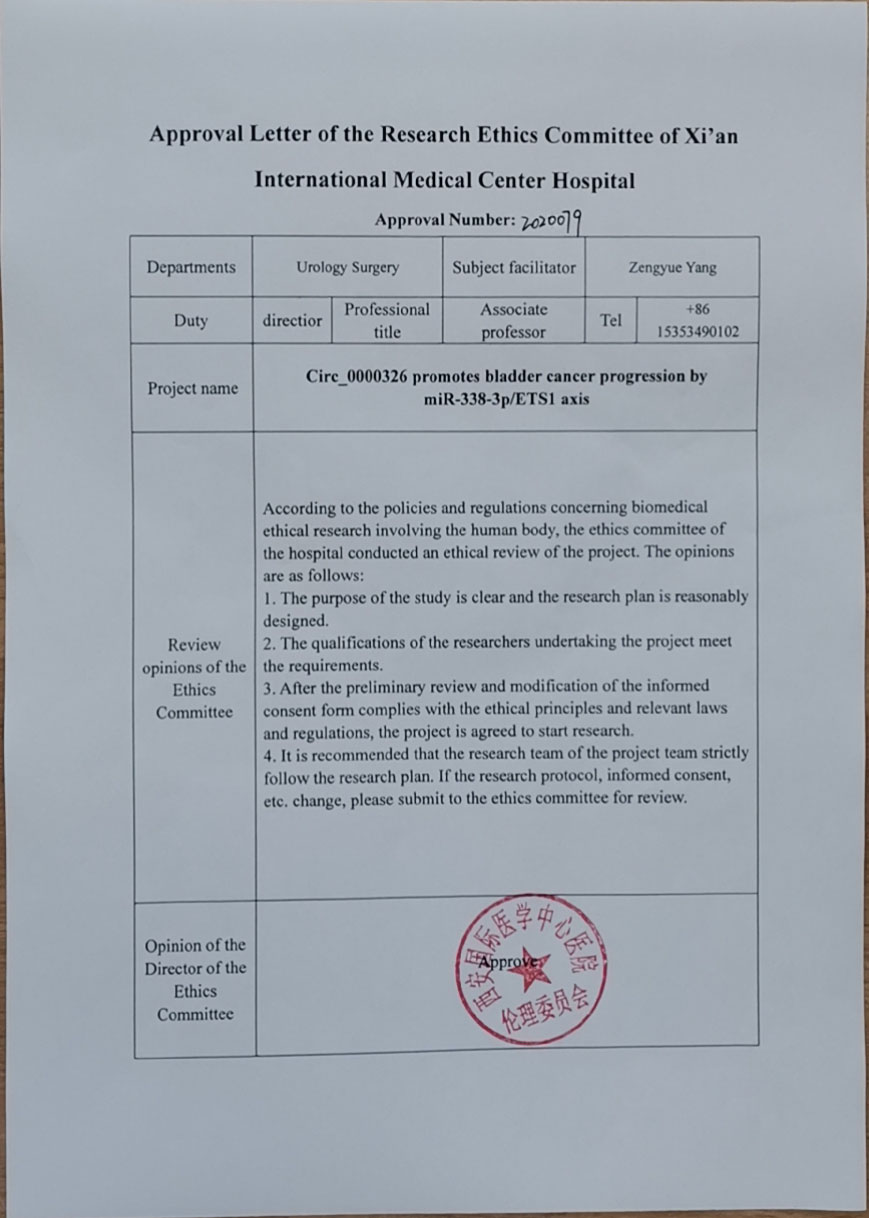


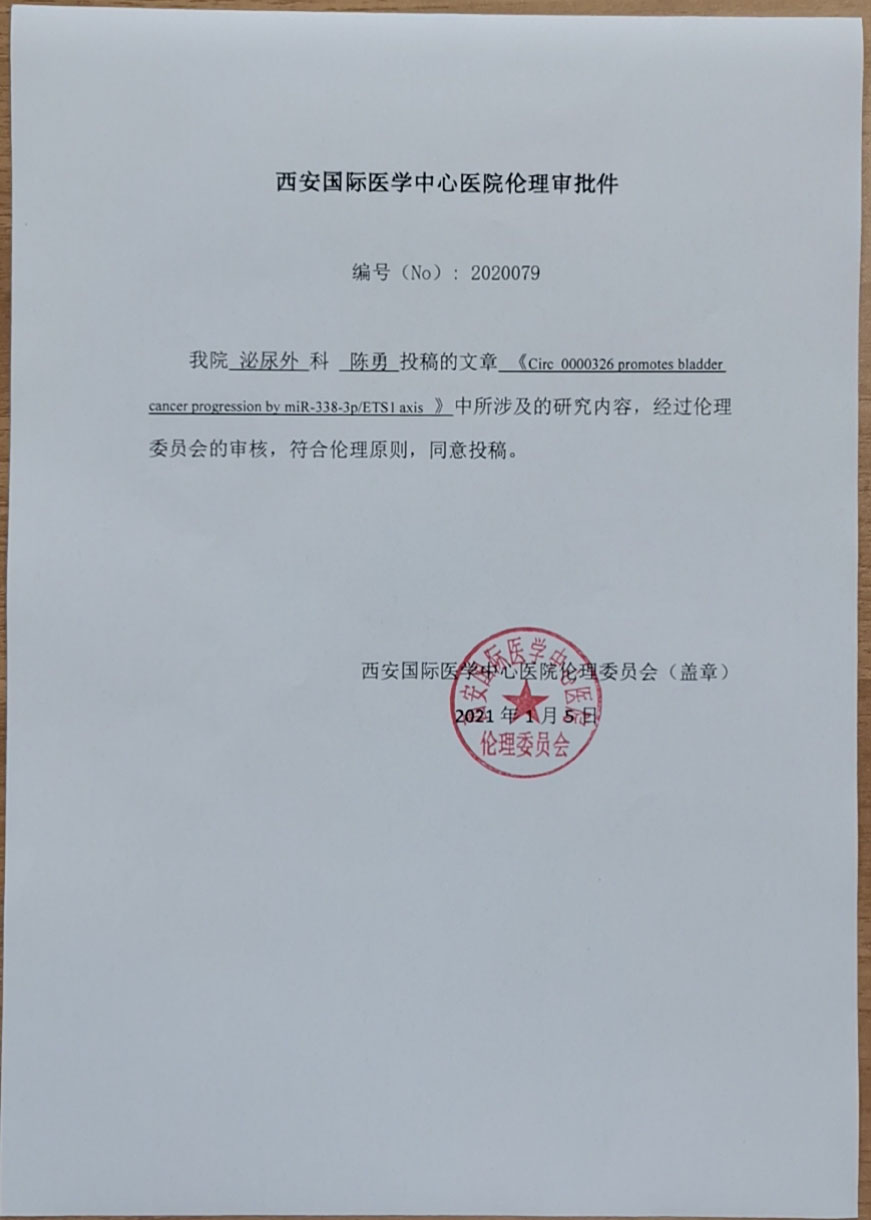

Supplement: Supplemental Material [file KBIE_A_2008738_SM3836.docx]
